# Entrustable professional activities versus competencies and skills: Exploring why different concepts are often conflated

**DOI:** 10.1007/s10459-022-10098-7

**Published:** 2022-02-28

**Authors:** Olle ten Cate, Daniel J. Schumacher

**Affiliations:** 1 Utrecht Center for Research and Development of Health Professions Education, University Medical Center Utrecht, P.O. Box # 85500, 3508 GA Utrecht, The Netherlands; 2 Cincinnati Children’s Hospital Medical Center, University of Cincinnati College of Medicine, Cincinnati, Ohio USA

**Keywords:** Entrustable professionals activity, Skill, Competency, Entrustment decision

## Abstract

Despite explanations in the literature, a returning question in the use of entrustable professional activities (EPAs) is how to distinguish them from competencies and skills. In this article, we attempt to analyze the causes of the frequent confusion and conflation of EPAs with competencies and skills, and argue why the distinction is important for education, qualification and patient safety. ‘Tracheotomy’, ‘lumbar puncture’, ‘interprofessional collaboration’ for example are colloquially called ‘skills’, but its is a person’s *ability* to perform these activities that is the actual skill; the EPA is simply the activity itself. We identify two possible causes for the confusion. One is a tendency to frame all educational objectives as EPAs. Many objectives of medical training can be conceptualized as EPAs, if ‘the ability to do X’ is the corresponding competency; but that does not work for all. We offer ways to deal with objectives of training that are not usefully conceptualized as EPAs. A more fundamental cause relates to entrustment decisions. The permission to contribute to health care reflects entrustment. Entrustment decisions are the links or pivots between a person’s readiness for the task and the actual task execution. However, if entrustment decisions do not lead to increased autonomy in the practice of health care, but only serve to decide upon the advancement to a next stage of training, EPAs can become the tick boxes learners feel they need to collect to ‘pass’. Gradually, then, EPAs can loose their original meaning of units of practice for which one becomes qualified.

## Introduction

A returning question in discussions about the definition and use of entrustable professional activities (EPAs) for educational purposes is how to distinguish them from competencies and skills. This is not a new question, and the distinction has regularly been stressed (ten Cate & Taylor, 2021; ten Cate et al., 2015), yet confusions keep arising.(Melvin et al., 2020) The distinction is key, not only to using EPAs and competencies separately and correctly but also to valuing their complementary nature.

Our aim in this paper to shed light on this distinction, to analyze the causes of frequent confusions and conflations and to detail why the distinction is important for education, qualification and patient safety.

### Competencies and skills: Attributes of individuals

Competencies, by their nature, need a context to make them visible. Imagine walking across the street and encountering three unknown individuals. They seem to vary in age from about 30 to 40. You cannot see their competencies, but one is a pianist, one is a computer scientist, and one is a neurosurgeon. Besides that, one is an advanced chess player, one a proficient wind surfer, and the third fluent in Arabic. You know nothing of this all by just encountering them on the street, which is not a concert hall, operating room, beach, tournament hall, or foreign country, which could have led you to guess those competencies. In short, they possess competencies that are not immediately visible. A competency, according to the dictionary, is ‘the ability to do something successfully or efficiently’ (NN, n.d.). It is *ability* that counts. An ability is a feature of an individual; competencies do not exist outside individuals. Competencies are typically developed through knowledge acquisition and practice experience, often dominated by cognition. Competenc*ies* are specific components of overall competence, suitable for specific tasks. The general consensus in the literature, and a useful way to conceive of them, is that they constitute the integration of knowledge, skills and attitudes (KSAs) that are required to perform those specific tasks.(van Merriënboer et al., 2002) However, this general consensus is not always followed in every-day use of the word competency. For example, the Accreditation Council for Graduate Medical Education (ACGME) and American Board of Medical Specialties in the United States originally included ‘medical knowledge’ and ‘patient care’ in their six core competencies (later correctly relabeled as ‘domains of competence’ (Carraccio et al., 2004)).

Skills, in everyday language, are often equated with competencies. That is not surprising, as the Oxford dictionary calls them ‘capability of accomplishing something with precision and certainty’—hardly distinguishable from competency. Many educators would agree that competencies combine skills, knowledge and attitude, suggesting that skills should differ from competencies, i.e. not include knowledge and attitude. But it is hard to envision performing a skill without procedural knowledge, and probably even some attitude, such as motivation to perform the skill well. Indeed, Dreyfus & Dreyfus, in their seminal work on skill development, maintain that skill includes, or even *is*, ‘knowing how’ to do something (Dreyfus & Dreyfus, 1986, p16) and without ‘knowing what’ (declarative knowledge), skill would probably not exist. Bloom’s famous distinctions of knowledge, skills and attitude (the cognitive, psychomotor and affective domains of educational objectives (Bloom et al., 1956)) may be somewhat artificial. These constructs (competencies, knowledge, skills, and attitude) are all attributes of individuals, present but invisible until they are being used. Their presence can only be inferred from observing the individual do something, such as carrying out tasks or activities or completing a test.

### Entrustable professional activities: Not attributes of individuals, but work to be done

Unlike competencies, activities are not qualities of people. Rather, they are work to be done. If a competency is the ability to do something successfully, the activity is that ‘something’. Execution of an activity requires specific competence (a competency or skill), but that activity in and of itself is not a competency or skill. EPAs, more specifically, are units of professional practice (tasks or bundles of tasks) that can be fully entrusted to an individual, once they have demonstrated the necessary competence to execute them unsupervised.(ten Cate & Taylor, 2021; ten Cate, 2005) The task, to be ‘entrustable’, ‘professional’, and ‘activity’, must meet additional conditions (have a beginning and end; be a stand-alone activity that is specific, observable, and restricted to qualified personnel; yield a recognized outcome of labor; and be suitable for an entrustment decision by a legitimate authority (ten Cate, 2005; ten Cate & Taylor, 2021)). They are the constituent activity components (units) of the practice of professionals; thus work, no matter who does it.

These contributions to practice must be done by legitimate members of the professional workforce, including peripheral members still in training, but qualified for these tasks.(Lave & Wenger, 1991) In any vocational education, practice starts within the educational program. Here is where EPAs become significant. Legitimate means that the trainee is accepted and sanctioned by a legitimate authority to perform that EPA. That could be a department head, or delegated to a professional with a supervisory role, and the decision should be backed by the judgment of an expert, preferably a team or committee. The synthetic nature of EPAs reflects the integrated fashion in which various competencies need to be applied simultaneously for adequate task execution.(Pangaro & ten Cate, 2013).

## How competencies and skills are often used for activities

Clearly, one cannot *possess EPAs*, nor become *qualified for competencies*. Rather, possessing competencies and being qualified for EPAs is the goal of medical training. Yet, in practice these are sometimes mixed up. That is not fully surprising. Common language use does not always abide by the logical distinctions we have tried to elaborate in the previous paragraphs. Popular use, particularly of ‘skill,‘ often reduces it to the activity only, instead of the *ability* to carry out that activity. Listings of relevant skills for health care often read to include things like ‘arterial line insertion’, ‘tracheotomy’, ‘communication’, ‘lumbar puncture’, ‘interprofessional collaboration’. The use of skill for activity is so ingrained in the educational language, and so hard to replace by different wordings, that advocating a different use would not likely succeed. However, using competency and skill for activities has also led some authors to envision EPAs as large, holistic skills or competencies.(Kappy et al., 2021) But if EPAs were just large competencies, how would one envision being entrusted with a competency?

**Fig. 1:**
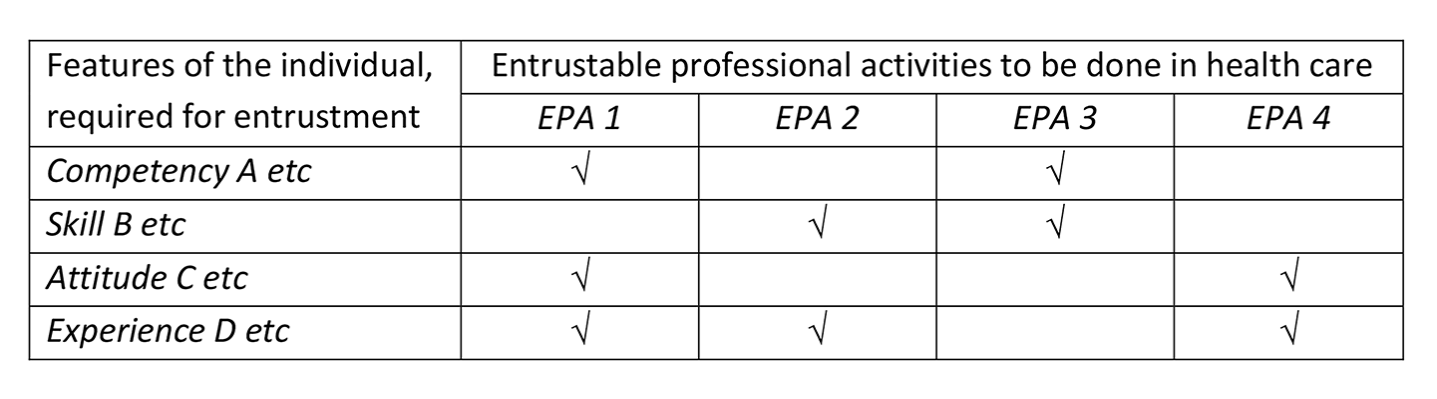
The two-dimensional representation of competencies/skill/abilities versus EPAs

That representation may perpetuate confusion. While EPAs require individuals to posses features that range from small microskills to large domains of competence, an EPA is not such a feature. EPAs can be assigned and executed, but they cannot be ‘possessed’ by individuals. These considerations are not on the same dimension. Rather, EPAs and competencies relate to each other as two separate dimensions (Fig. 1) (ten Cate et al., 2015; ten Cate & Scheele, 2007). Also, viewing the ability to perform an EPA as the “largest” unit of a competency or skills is not necessarily correct. Some EPAs are not particularly “large” activities. For example, EPAs for medical students are smaller in their breadth or responsibility than EPAs for residents and fellows.

Using EPAs and assessing the ability to perform them should change the focus of assessment to making a *decision* about whether or not to entrust someone with the responsibilities of an activity, at a specified level of supervision, or at least producing a recommendation for such a decision (ten Cate et al., 2020). Transfer of responsibilities to trainees affects the quality and safety of patient care and requires thoughtful consideration.

## Causes for the confusion

We believe that there are at least to two causes for the confusion seen between EPAs and competencies and skills. One is a tendency to translate all objectives of medical training into EPAs. The other is the use of entrustment decisions not for entrustment with health care tasks, but rather for progression in training.

### Dealing with educational objectives that do not fit the definition of an EPA

Some skills equate to activities that would not qualify to be EPAs. To use an example from the introduction above, one of the three passengers possesses the skill of speaking and understanding Arabic. “Speaking Arabic” could be a skill or activity, but it would not per se make for an EPA. “Acting as an interpreter” for a patient, or for a politician in an international diplomacy meeting, would, however, be an EPA, because it is a contribution to professional work (i.e. it is something that must be done, and someone must be hired and relied on), and it requires legitimate entrustment. The ability (skill, competency) to speak Arabic is of course the most important prerequisite to be trusted as an interpreter, but confidentiality, integrity, and reliability may weigh in. Indeed ‘observing confidentiality, ‘showing integrity’, ‘being reliable’ would also not fit with the definition of an EPA. For undergraduate medical education, Meyer et al recently concluded that among the US Core EPAs for Entering Residency (Englander et al., 2014) some do not meet the criteria to be an EPA (Meyer et al., 2020; Taylor et al., 2017), including ‘Collaborate as a member of an interprofessional team’ and ‘Identify system failures and contribute to a culture of safety and improvement’. There is little discussion that interprofessional collaboration and contributing to patient safety are essential, but capturing these in specific EPAs is problematic (ten Cate & Pool, 2020). Likewise, aspects of professionalism do not work well as EPAs. The further these features are distanced from concrete activities, the more difficult it becomes to qualify them as EPAs. Educators may maintain that knowledge of the history of medicine is essential for adequate performance, or that without a deep understanding of social injustices in society related to access to medical care, ethical care provision is not possible. Or that knowledge of the humanities (art, literature, philosophy), or international experience or having done a research project is helpful to become a better doctor.

The questions then becomes: if the profession is defined by its activities, how should we relate these features to EPAs? We offer three ways to think about this question.

*Through specification of EPAs.* Interprofessional collaboration may include ‘chairing interprofessional meetings’. That activity meets the criteria of an EPA. ‘Investigating a patient safety incident’ (including the completion of a report with recommendations) could be a specification of ‘contributing to a culture of safety’. If these activities can meet the criteria of EPAs (ten Cate, 2005), they could be a suitable for true entrustment decisions.

*Through inclusion in assessment criteria of EPAs.* A second way is to consider the feature merely as a quality of the individual that is *conditional* for entrustment decisions for one of more EPAs. Full EPA descriptions (ten Cate & Taylor, 2021) include a section of required knowledge, skills, attitudes and experiences that are conditional for entrustment decisions. Task-specific trustworthiness (Schumacher et al., 2021) requires that a trainee attends to all contextual aspects of the task, including, for example, the interprofessional collaborative context. This is likely an excellent example of something conditional for entrustment to perform any EPA. In surgery, many EPAs that can be listed as surgical procedures in the operating room would require adequate interprofessional communication and collaboration during surgery (such as with scrub nurses, anaesthesiologists, anaesthesia technicians). But, interprofessional collaboration is not an EPA in itself. The competencies-EPAs matrix (ten Cate et al., 2015; ten Cate & Scheele, 2007) as depicted in Fig. 1 can include any relevant feature. Performing EPAs requires the integration of several of these features, and are thus holistic in nature.

*Objectives not linked to EPAs*. A third option is to regard some objectives as general requirements for education and graduation, not for particular EPAs. Such objectives can be considered important but not conditional for entrustment decisions. Physicians, as mature and responsible citizens, may be expected to have thoughtful and nuanced opinions, not only about health care related issues, but also about politics, ethics, management, economics, history, climate change, racism, gun control, humanities. These general qualities arguably make them better physicians, but they cannot easily be captured in individual EPAs. One may argue that this category is not essentially different than category 2, as these general features may improve the performance in EPAs. That is true, but when they are not *conditional* for EPA entrustment decisions (i.e. to be measured and marked as satisfactory in some way), *and* if it is difficult to determine for which EPAs they are important and for which they are not, it is better to regard these features as a third, separate category: important but not necessarily conditional for EPA entrustment decisions. Not everything that is important for physicians can or must be an EPA.

### Entrustment decision making without consequences for clinical responsibility

This cause of the conflation of EPAs with skills or competencies is more fundamental. When rules and regulations, often fueled by patient safety or liability concerns, prescribe that all patients must be seen by a consultant specialist, as long as the trainee physician has not yet completed training, there is a risk that entrustment decisions lose their significance. In other words, someone could be deemed ready for entrustment with unsupervised practice but required to have some level of supervision on account of not yet meeting a requirement in place from a regulatory standpoint. Consider the Arabic interpreter example above. Someone may be fluent in Arabic and trusted to serve as an Arabic interpreter by a training program but unable to actually do this in practice because the institution requires them to pass a test before doing this. Such limitations abound in healthcare where trainees are entrusted to perform unsupervised practice but must still be supervised because training requirements mandate that.

These situations decrease trainees’ opportunities to develop autonomy (Halpern & Detsky, 2014) and can lead to undue ‘seniorization’ of health care tasks to supervisors, which neither benefits trainees (Dacey & Nasca, 2019) nor patients.(Kunac et al., 2021) They can also decrease the urgency for thoughtful assessment of readiness for entrustment during training and increases the need for supervision after training, because trainees are left without the experience of full autonomy and responsibility at the time of graduation.(Mattar et al., 2013; Turner et al., 2021) If entrustment decisions do not lead to a new status of autonomy for an EPA, even if they are made with strong validity evidence (Touchie et al., 2021), entrustment “decisions” become a paper exercise. Complaints that EPA’s become tick-box exercises for learners and assessors because entrustment decisions have lost their meaning (or never acquired their true meaning) easily explain why EPAs then only serve to acknowledge competence and then *become* a competency. In the current Canadian Competence-By-Design (CBD) model, summative entrustment decisions are made to promote trainees from one stage of training to the next rather than considering a decrease in supervision for an EPA. If “entrustment decisions” just lead to progression to a next stage, they serve as endpoints for activities instead of beginning points of increased autonomy for these activities. A true entrustment decision should entitle a learner to become a genuine, unsupervised team member. In CBD, EPAs are mandatory, but do not appear to be the building blocks that allow for greater autonomy after a summative decision. [https://www.royalcollege.ca/rcsite/cbd/implementation/cbd-milestones-epas-e]. Hence, the power of EPAs to operationalize CBME is not fully exploited; evaluations remain retrospective and assessment scales focus on proficiency, rather than on future-focused recommendations for actual entrustment with greater responsibility. Within that context it is not surprising that EPAs are felt as competencies, that must be ticked off, to allow leaving them behind.

In contrast, the opposite can be true. In some programs, entrustment decisions are not deliberately made because there is an assumption that a learner at a specific duration in training is ready for increased responsibilities by default, even without valid assessment evidence to support this. Melvin et al. report how, in Internal Medicine, residents having reached a particular stage, are presumed to be able to function in that role. Because the system has thus already predetermined entrustment, attendings do not actively make entrustment decisions.(Melvin et al., 2020) This practice is at variance with the purpose of competency-based education, where fixed duration and variable standards are to be replaced by fixed standards and, if needed, variable duration.(Carraccio et al., 2002; Frank et al., 2010; McGaghie et al., 1978). In undergraduate programs, entrustment decisions are often also theoretical, reflecting an intention to trust or an decision on trustworthiness, rather that an actual entrustment decision (Brown et al., 2021; Geraghty et al., 2021; Postmes et al., 2021). This leaves the power of entrustment decisions underutilized and EPAs may subsequently become just educational objectives.

Finally, it is not always possible to grant trainees the autonomy that matches their competence, even if established with strong validity evidence. Unlicensed students will always require direct or indirect supervision, no matter how impressive their performance, and junior residents may be more autonomous but will need to report to responsible supervisor. The challenge is to create an environment in which supervision is close enough to be safe, yet feels distant enough to fuel the responsibility that pushes a learning curve.(Babbott, 2010; ten Cate, 2018). Surgical residents, for example, perceive trust when they find that their pre-operative plan is no longer being corrected, a useful measure of trust.(Kearse et al., 2021). Building trust and valid entrustment decisions require acquaintance with trainees. Short rotations and a lack of continuity of supervision may therefore also be a reason for true entrustment decisions not to occur.

## In conclusion

We hope to have shed slight on the essential distinction between EPAs (activities; units of work) and the conglomerate of competencies and skills, (qualities of persons), and on the causes of the frequent conflation of these constructs. Three lessons stand out: (1) the ability to complete an EPA is a quality of a person but the EPA itself is an activity, (2) the full power of entrustment decisions can only be achieved if they have consequences for the autonomy and responsibility of trainees in health care, and are not limited to decisions about progression to a next phase of training or, even worse, paper exercises without any consequence to them at all, and (3) not all objectives of medical education can be captured in EPAs. With these in mind, there may be less tendency to conflate EPAs with competencies.
